# Hepatitis A, Italy

**DOI:** 10.3201/eid1107.041157

**Published:** 2005-07

**Authors:** Raffaele D'Amelio, Alfonso Mele, Andrea Mariano, Luisa Romanò, Roberto Biselli, Florigio Lista, Alessandro Zanetti, Tommaso Stroffolini

**Affiliations:** * Direzione Generale della Sanità Militare, Rome, Italy;; † Università degli Studi "La Sapienza" II Facoltà di Medicina e Chirurgia, Rome, Italy;; ‡ Istituto Superiore di Sanità, Rome, Italy;; § Istituto di Virologia, Università degli Studi, Milano, Italy;; ¶ Centro Sperimentale di Volo, Pratica di Mare, Italy;; # Centro Studi e Ricerche di Sanità e Veterinaria, Rome, Italy;; ** Ospedale S. Giacomo, Rome, Italy

**Keywords:** HAV, Italy, Seroprevalence, Epidemiology

**To the Editor:** Hepatitis A virus (HAV) infection rates are very low in industrialized countries. A noticeable fall in the prevalence of HAV antibodies (anti-HAV) has been reported in southern European and Mediterranean countries such as Spain (*1*) and Greece (*2*), reflecting improvements in hygiene standards in the last decades.

An HAV prevalence of 66.3% in 1981 (*3*) and 29.4% in 1990 (*4*) was shown in studies conducted in military recruits from all Italian regions. In both studies, subjects from southern regions had a higher HAV prevalence than those from north-central regions. In 2003, we conducted a study of recruits to show changes in HAV infection prevalence in younger Italian generations.

Military service was compulsory in Italy at that time; all men 18–26 years of age were included. From September to December 2003, 323 recruits 18–26 years of age (mean age 20 years), representing all Italian regions, who had been accepted for Air Force military service were tested for anti-HAV in the recruitment center at Viterbo. This recruitment center, used in the 1990 study, was chosen again because it is located near Rome and adherence to protocol was easier to control.

A standard, precoded questionnaire was designed to collect information in the same sequence as questions asked by military personnel during the examination. The same information was collected as in the previous studies: date of birth, residence, father's years of education, and family size. After informed consent was obtained, blood samples were collected and stored at –30°C until tested. No person was vaccinated against HAV. Anti-HAV assay was performed by using commercial immunoenzymatic method (Abbot Laboratories, North Chicago, IL, USA). The methods used in the 1981 and 1990 studies have similar sensitivity and specificity between them and in relation to that used in the current study, and are detailed elsewhere (*3**,**4*).

The prevalence of anti-HAV declined from 66.3% in 1981 to 5.3% in 2003 (p<0.01, χ^2^ test). In 2003, the prevalence was 2.1% in the north-central region and 7.9% in southern regions (Figure). However, southern residents were more likely to have been exposed to HAV than north-central residents (p<0.02, χ^2^ test). No statistical difference relative to father's years of education or family size was shown. Basic requirements for Navy (1981 study) and Air Force (1990 and 2003 studies) enrollment were similar. Thus, the 3 studies are comparable and a valid estimation of epidemiologic changes over time.

**Figure:**
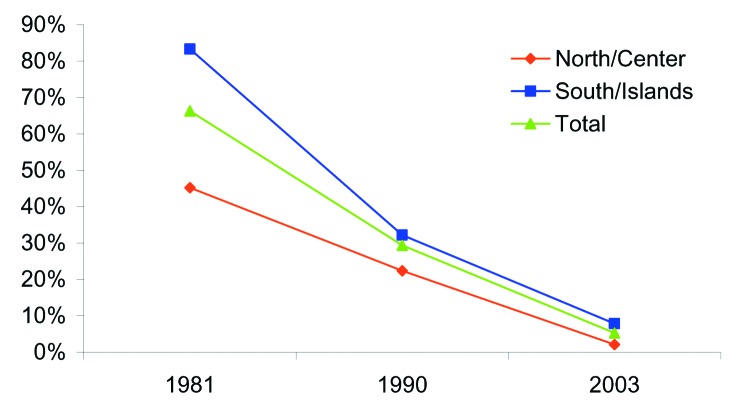
Anti-hepatitis A virus prevalence, 1981–2003.

The anti-HAV prevalence shown in this study (5.3%) indicates that Italy has very low endemicity of HAV infection, at least in the young male population. The decline of HAV infection for >20 years is a consequence of good sanitation and hygienic conditions (vaccination against HAV is rarely performed in Italy) and has generated an increasing proportion of adults who are susceptible to this virus at an age characterized by the likely occurrence of a more severe clinical illness (*5*). This situation will likely necessitate costly interventions, such as vaccinating risk groups (e.g., military personnel, healthcare workers), to prevent HAV infection. Thus, HAV vaccination has been included in the compulsory vaccination schedule of the Italian military personnel since 1998 (*6*).
